# Diet and Food; Considered in Relation to Strength and Power of Endurance, Training and Athletics

**Published:** 1899-04

**Authors:** 


Diet and Food; Considered in Relation to Strength and Power of Endurance, Training and Athletics. By Alexander Haig, M. A. and M. D., Oxon., F. R. C. P. With five illustrations. Pages 86. London: J. & A. Churchill, 7 Great Marlborough Street, W. 1898. Philadelphia: P. Blakiston's Son & Co.

The author felt compelled to write these few pages because of the large amount of ignorance and prejudice prevailing in regard to the questions of diet and body strength and nutrition; also because it will be found that in the diet lies the key to nine-tenths of the social and political problems that vex our nation and time.

"Diet, as at present used, is often the product of a vast amount of ignorance; it is the cause of a hideous waste of time and money; its produces mental and moral obliquities, destroys health and shortens life and generally quite fails to fulfil its proper purpose." It will thus seem to be a very timely topic for consideration and one that deserves a broad and wide field for discussion. The ideas of the author so well known by the profession from his work on uric acid are forcibly and ably expressed in this little volume and are well worth perusal, even if the reader cannot concur in all that the author presents. To banish all meat foods, eggs and the lighter stimulants, as the coffee and cocoa, and to live on the vegetables, fruits, carbohydrates may do in certain climates, but in such a changing climate, such as we live in, it must seem that a mixed animal and vegetable diet would be preferable. The author's way of explaining the deleterious effects of a flesh diet is interesting and instructive and the same method pervades the whole work.

The book is beautifully gotten up and is an ornament to any physician's library. W. C. K.

